# Motor precision shapes information seeking in perceptual decisions

**DOI:** 10.3758/s13423-026-03038-8

**Published:** 2026-11-06

**Authors:** Alessandra Loretani, Claire Bédé, Thibaut Gajdos Preuss, Andrea Desantis

**Affiliations:** 1 Département Traitement de l’Information et Systèmes, ONERA, Salon-de-Provence, France; 2 Institut de Neurosciences de la Timone (UMR 7289), CNRS and Aix-Marseille Université, Marseille, France; 3 Centre de Recherche en Psychologie et Neurosciences (UMR 7077), CNRS, Aix Marseille Université, Marseille, France; 4 Integrative Neuroscience and Cognition Center (UMR 8002), CNRS and Université Paris Cité, Paris, France

**Keywords:** Information-seeking, Confidence, Decision making, Motor cost

## Abstract

Adaptive behavior relies heavily on the confidence we have in our decisions. For instance, in the absence of explicit feedback, the confidence associated with a decision determines whether we seek additional evidence or are likely to change our mind. Recent studies suggest that confidence estimation also depends on motor processes involved in implementing perceptual decisions. However, whether motor-induced changes in confidence meaningfully affect behavior, such as by altering information-seeking, remains uncertain. The present study addresses this question. Participants performed a perceptual task requiring discrimination of the orientation of Gabor patches. The motor cost of the perceptual response was manipulated by varying the size of the response circles that participants had to reach to report their judgment. Consistent with previous findings showing that easier motor responses are associated with lower confidence ratings, we found that such responses were also linked to a greater tendency to seek additional evidence compared to more costly responses. Importantly, this increase in information-seeking behavior was not accompanied by any change in perceptual accuracy. These findings support the idea that the motor costs associated with perceptual decisions modulate confidence, and that such motor-induced changes in confidence play a functional role in guiding subsequent behavior.

**Supplementary Information:**

The online version contains supplementary material available at https://doi.org/10.3758/s13423-026-03038-8.

## Introduction

Sensory information is uncertain and noisy, yet humans are capable of evaluating this uncertainty and estimating how confident they are that their perceptual decisions are correct. Research showed that this metacognitive process plays a critical role in adapting and guiding behavior in the absence of external feedback (Rahnev et al., 2022). For example, confidence contributes to learning (Guggenmos et al., 2016), guides information seeking (Desender et al., 2018; Pescetelli et al., 2021) and change of mind (Rollwage et al., 2020; Sanchez et al., 2024b), and is also crucial in social interactions (Bahrami et al., 2010).

Perceptual confidence relies primarily on the quality of sensory evidence (Kiani & Shadlen, 2009), but it is also influenced by processes involved in the execution of the action used to implement the decision (Fleming et al., 2015; Gajdos et al., 2019; Sanchez et al., 2024a, 2025; Siedlecka et al., 2021; Turner et al., 2021; Wokke et al., 2020; but see also Hagura et al., 2023). For example, Sanchez et al. (2024a) showed that when the motor cost associated with a perceptual response was higher, confidence increased, even though accuracy remained unchanged. These findings suggest that motor-related signals involved in the execution of a perceptual decision feed confidence, in line with recent accounts on which perceptual confidence integrates multiple sources of information beyond the sensory evidence itself (Fleming & Daw, 2017; Mamassian & de Gardelle, 2022). The involvement of motor signals in perceptual metacognitive evaluation may seem counterintuitive: if individuals already have access to the quality of their sensory evidence, why rely on motor cues? However, this idea becomes less counterintuitive when considering that motor signals may convey information linked to first-order accuracy: for example, the degree of commitment to a decision (cf. Turner et al., 2021). In fact, more broadly, confidence judgments may rely on heuristics that employ cues other than the quality of sensory evidence (Ackerman, 2019; Allen et al., 2016; Kiani et al., 2014; Maniscalco et al., 2016; Shekhar & Rahnev, 2024; Van Marcke et al., 2024).

One critical question that remains to be answered is whether motor-induced changes in perceptual confidence genuinely reflect the metacognitive evaluation of the perceptual decision, or whether they instead depend on the way confidence is reported. The effects described above have been established almost exclusively through explicit confidence reports, obtained by asking participants how confident they are that their response was correct. Yet explicit confidence judgments may be biased by different forms of information (Mamassian, 2016; Rahnev & Denison, 2018). In these situations, when judging whether their response was correct, participants might conflate the quality of their perceptual representation (e.g., “how well did I see the stimulus?”) with the quality of their response execution (e.g., “did I perform the movement well/correctly?”). The reported influence of motor signals on confidence might therefore partly reflect the way confidence is probed, rather than a genuine contribution of motor information to the metacognitive evaluation of the perceptual decision.

If, on the contrary, motor signals genuinely inform observers’ assessment of the quality of their perceptual representation, their influence should extend beyond explicit confidence reports. It should also be visible in metacognitively guided behaviors that do not require an explicit confidence judgment, such as information seeking. Observing such an influence would indicate that action-induced changes in confidence are not merely epiphenomenal by-products of the reporting procedure, but play a functional role in shaping subsequent behavior aimed at minimizing uncertainty. The present study aimed to address this question. As in Sanchez et al. (2024a) participants discriminated the orientation of a Gabor patch, and we manipulated the cost associated with executing their perceptual response. After their decision, participants could choose to view the Gabor again to improve their chances of reporting the correct orientation. Consistent with Sanchez et al. (2024a), who found that confidence increased when perceptual responses were more costly, we observed that information seeking decreased when responses were more costly, while accuracy remained unaffected. This suggests that action-induced changes in confidence functionally shape subsequent behaviors such as information seeking.

## Materials and methods

### Participants

Given that this experiment reproduced the same method as Sanchez et al. (2024a), we fixed the sample size at 24 adult participants, as in that study. A total of 29 adults took part in this experiment in return for a 10 € gift card. All participants had normal or corrected-to-normal vision, and were naïve to the hypothesis under investigation. This study was conducted in agreement with the requirements of the Declaration of Helsinki and was approved by the ethics committee of Université Paris Cité (N° IRB: IORG0010120-2025-77). Five participants were excluded from the sample: two because they did not complete the task, and three because they did not meet the minimum performance criterion, set at 55% average accuracy across all conditions. The final sample consisted of 24 participants (17 males; average age = 28.6 years,^1^*SD* = 6.3 years).

### Apparatus

Stimuli were presented on an LCD monitor (53 cm wide) with a refresh rate of 60 Hz and a resolution of 1,920 × 1,080 pixels. Stimulus presentation and data collection were performed using Python and the Psychopy software (Peirce et al., 2019). Participants responded with a mouse at 800 DPI. Head movements were restrained with a chinrest located 70 cm from the screen.

#### Stimuli and procedure

Participants completed eight blocks consisting of 24 trials each, for a total of 192 trials. Each trial started with a white central fixation cross (with a height and width of 0.4 degrees of visual angle (dva)), displayed at the center of the screen for 300 ms. After this delay, a vertical or horizontal Gabor patch (1.8 dva diameter and 1.11 cycles per dva) was presented for 50 ms within a noise texture, and participants reported its orientation. The contrast of the Gabor was set to a value supporting a proportion of 0.75 correct discrimination performance, calculated separately for each participant in a preliminary calibration phase (see below).

**Fig. 1:**
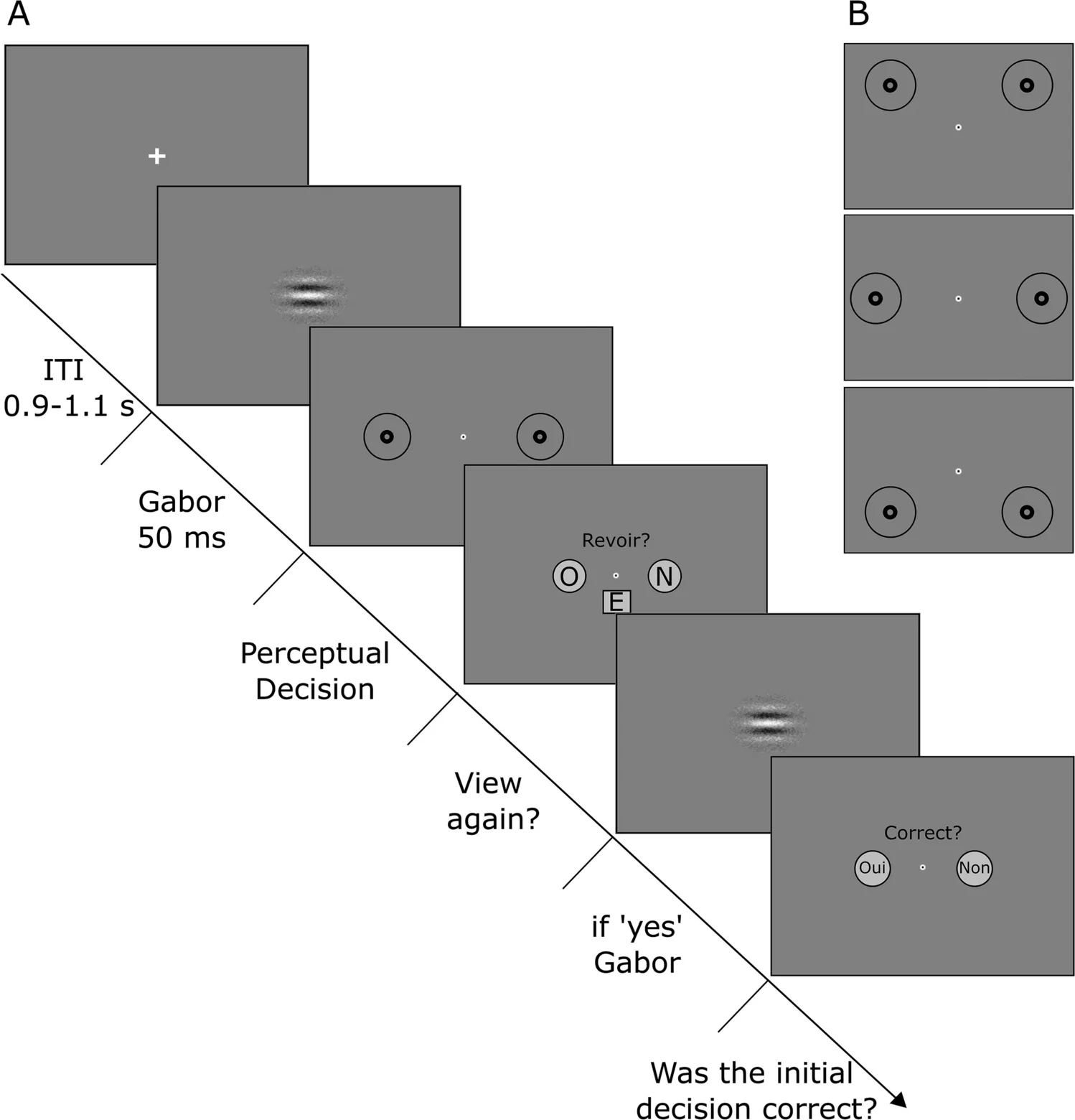
Schematic illustration of a trial. The images are for illustrative purposes and are not drawn to scale. (**A**) The trial sequence began with the presentation of a white cross. Subsequently, a Gabor patch was displayed. At Gabor offset, two concentric circles (a small circle within a big one) were presented on either side of the screen, along with a white cursor at the center. Participants reported the orientation of the Gabor by clicking on either a left or a right response circle (e.g., the left circle for horizontal, and the right circle for vertical). In each trial, either the small or the big circle had a thicker contour, indicating the set of circles that participants needed to reach to report their perceptual decision (in this example, the small circles). After the perceptual decision, participants decided whether to see the stimulus again. An “error” rectangle was displayed at the bottom of the fixation, allowing participants to report any unintentional errors in their perceptual response. If participants decided to view the stimulus again, the Gabor was presented once more, and they reported whether their initial guess was correct or not. (**B**) The three possible locations for the response circles: top, middle, and bottom, each equidistant from the center of the screen

At Gabor offset, two concentric circles, a small circle (0.3 dva radius) inside a large one (2.3 dva radius), were displayed on both sides of the fixation (Fig. 1). Each side corresponded to a specific orientation of the Gabor (e.g., left for horizontal and right for vertical). In low precision trials, the outline of the big circles was thicker, indicating that the orientation of the Gabor had to be reported by clicking within either the left or the right *big* circle. In high precision trials, the outline of the small circles was thicker, indicating that participants were to report the Gabor’s orientation by clicking within either the left or the right small circle. High and low precision trials were equiprobable and randomly distributed.

Once the mouse cursor moved to the left or the right and crossed an invisible vertical boundary located 3.67 dva away from the central vertical line of the screen, participants could not alter their response. In other words, they could not change their mind and go to the other side of the screen to select the other response circle. If they tried they would see the mouse cursor blocked by an invisible barrier located vertically at the center of the screen. This served two purposes. First, it ensured that involuntary mouse movements were not labelled by the script as a “response.” Second, it provided a defined moment of commitment to a perceptual decision.

In addition, to prevent repetitive and stereotypical movements, the concentric circles could appear equiprobably in one of three different locations on the screen (top, middle, or bottom, as illustrated in Fig. 1). The left and right concentric circles were equidistant from the central vertical line of the screen (at 15 dva from the center of the circles). Importantly, the mouse cursor was invisible at the beginning of the trial, and during Gabor presentation it appeared at the center of the screen when a perceptual response was required.

If a participant did not respond within 3 s (from Gabor offset), the screen displayed the message “Too late,” and the trial was reintroduced in a random position within the remaining trials of the block. The mapping of responses was counterbalanced among the participants. Half of them employed the circles on the right side of the screen to signify a vertical Gabor, while those on the left side indicated a horizontal Gabor. For the remaining half of the participants, the opposite mapping was employed.

Following their perceptual decision, participants were asked whether they wanted to view the Gabor again. They were explicitly instructed that they should use this opportunity to improve their performance. Two response circles were shown: one to the left and one to the right of fixation. Each circle contained a letter indicating the choice: “O” for Oui (yes in French) and “N” for Non (no in French). Participants indicated their choice by clicking the corresponding circle with the mouse. There was no time limit for this decision. Circles had a radius of 0.9 dva and were presented at an eccentricity of 3.24 dva (center-to-fixation). The spatial assignment (left or right) of the letter “O” and “N” was randomized across trials. Participants could also acknowledge an error in their initial perceptual decision by clicking a labeled “error” rectangle. These trials were not reintroduced into the trial list.

If participants chose to view the stimulus again, the Gabor was replayed with the same temporal dynamics. After the replay, participants indicated whether their initial guess was correct by clicking either the left circle labeled “C” (for “correct”) or the right circle labeled “I” (for “incorrect”; circle radius 0.9 dva, eccentricity 3.24 dva). We chose not to require a second perceptual judgment to avoid biasing the “view again” decision by the potential cost of repeating a fine motor action (i.e., reaching to small targets). The spatial assignment (left or right) of the letters “C” and “I” was randomized across trials. At the end of each trial, the mouse cursor was automatically repositioned to the screen center.

#### Calibration and training phases

Before starting the main experiment, we determined participants’ orientation discrimination thresholds using a triple interleaved staircase. This method allowed us to identify Gabor contrast values associated with a 75% orientation discrimination accuracy. Gabor visibility was manipulated by varying the nominal contrast of the grating (cf. the contrast parameter of psychopy GratingStim function, which scales the luminance modulation around the mean gray level), rather than through its opacity. Each staircase comprised 40 trials and the step size of each staircase was adjusted using an adaptive procedure known as accelerated stochastic approximation (Kesten, 1958). As for the main task, in the staircase phase, participants reported the orientation of the Gabor by moving the mouse within one of the two response circles (big or small). Response circles were randomized. During this phase they were not asked if they wanted to view the stimulus again.

Prior to the staircase, participants completed 60 staircase training trials in which they were familiarized with the stimuli and the perceptual task. After this training, participants performed 120 staircase trials (i.e., three interleaved staircases × 40 trials). Subsequently, they were familiarized to the primary task with 20 training trials. During this training we also verified whether the contrast value obtained with the staircase corresponded to the desired accuracy level. If not, the contrast was slightly increased or decreased by the experimenter depending on whether the performance was too low (below or equal to 60%) or too high (above or equal to 90%), respectively.

#### Behavioral analyses

Statistical analyses were conducted using hierarchical Bayesian models implemented with brms package (Bürkner, 2017) in R 4.4.1 (R Core Team 2024). We used Bayesian analyses because they are more robust to convergence issues and provide stable parameter estimates even with complex hierarchical structures or limited data compared to classical frequentist approaches (Kruschke, 2021). The precise model structure for the analyses was based on the pre-registered study of Sanchez et al. (2024a). We analyzed accuracy and “view again” responses using binomial models with a logit link function, and all timing variables related to participants’ perceptual response using ex-Gaussian models with an identity link. Trials in which participants acknowledged making an error in their initial perceptual decision were excluded from the analyses. They represented 2.22% of the total number of trials (57 in big circle trials and 43 in small circle trials).

Participants’ response time (RT) was defined as the entire movement latency, from Gabor onset to the click within the response circle. RT was divided into two intervals: commitment time (CT), the delay between Gabor onset and the moment the cursor exited the central zone (see *Method*), and execution time (ET), the interval between CT and the click within the response circle. We do not claim that CT and ET are pure estimates of a latent decision process, but they serve as behavioral proxies for two distinct moments of the response. CT indexed the commitment to a perceptual decision, capturing the initial movement latency (i.e., the decision to report a vertical or horizontal Gabor by moving left or right), while ET reflected the time needed to confirm that decision by bringing the cursor into the response circle. There are theoretical reasons to distinguish these latencies, and they guided the structure of our models with regard to accuracy and view-again responses (cf. Sanchez et al. 2024a). View-again responses occur after both CT and ET, so the two latencies may plausibly influence information seeking differently. For accuracy, only CT is relevant: once participants had moved the cursor out of the central zone, the response could no longer be changed (see *Method*), so nothing after CT could logically affect accuracy.

When RT, CT, and ET were used as continuous predictors, they were centered based on the mean across subjects. Categorical factors were coded using sum contrasts. Follow-up tests were computed with the emmeans package (Lenth & Piaskowski, 2025), and figures were generated with the ggplot2 package (Wickham, 2016).

Parameters were classified as showing strong evidence for an effect when their 95% credible intervals (CrIs) excluded the null value: zero for models with a continuous outcome (timing variables), and one for effects expressed as odds ratios (accuracy and view-again models). This heuristic follows the interpretation of the probability of direction proposed by Makowski et al. (2019).

## Results

### Commitment and execution time

We assessed the impact of accuracy (correct, incorrect), circle (big, small), position (top, middle, bottom), and their interaction on commitment times (ct ~ accuracy*circle*position + (1 + accuracy*circle*position | subject)). We observed evidence that commitment was faster for correct (*M* = 795 ms, *SD* = 281 ms)^2^ than incorrect decisions (*M* = 905 ms, *SD* = 310 ms; β = −19.95, CrI [−25.73, −14.74]). Neither circle (*M* big = 824 ms, *SD* = 299 ms; *M* small = 830 ms, *SD* = 290 ms; β = 2.43, CrI [−1.76, 6.63]) nor any other factor showed a credible effect on commitment time (Online Supplementary Material (OSM) Table 1S and Fig. 1S).

**Table 1:** Hierarchical Bayesian regression table predicting “view again” judgments from accuracy, circle, position, and commitment time and execution time. Commitment and execution time were mean-centered. We report only strong evidence in favor of a difference; full results are reported in the Online Supplementary Material (Table 8S)

	View again	
*Predictors*	*Odds ratios*	*CI (95%)*
accuracy_correct	0.67	0.58–0.76
circle_small	0.84	0.74–0.94
execution time	4.10	2.49–6.87
commitment time	81.23	22.75–327.74
accuracy_correct × commitment time	2.35	1.52–3.57
circle_small × position_middle × commitment time	0.57	0.33–0.96
N _subject_	24	
Observations	4,508	

**Fig. 2:**
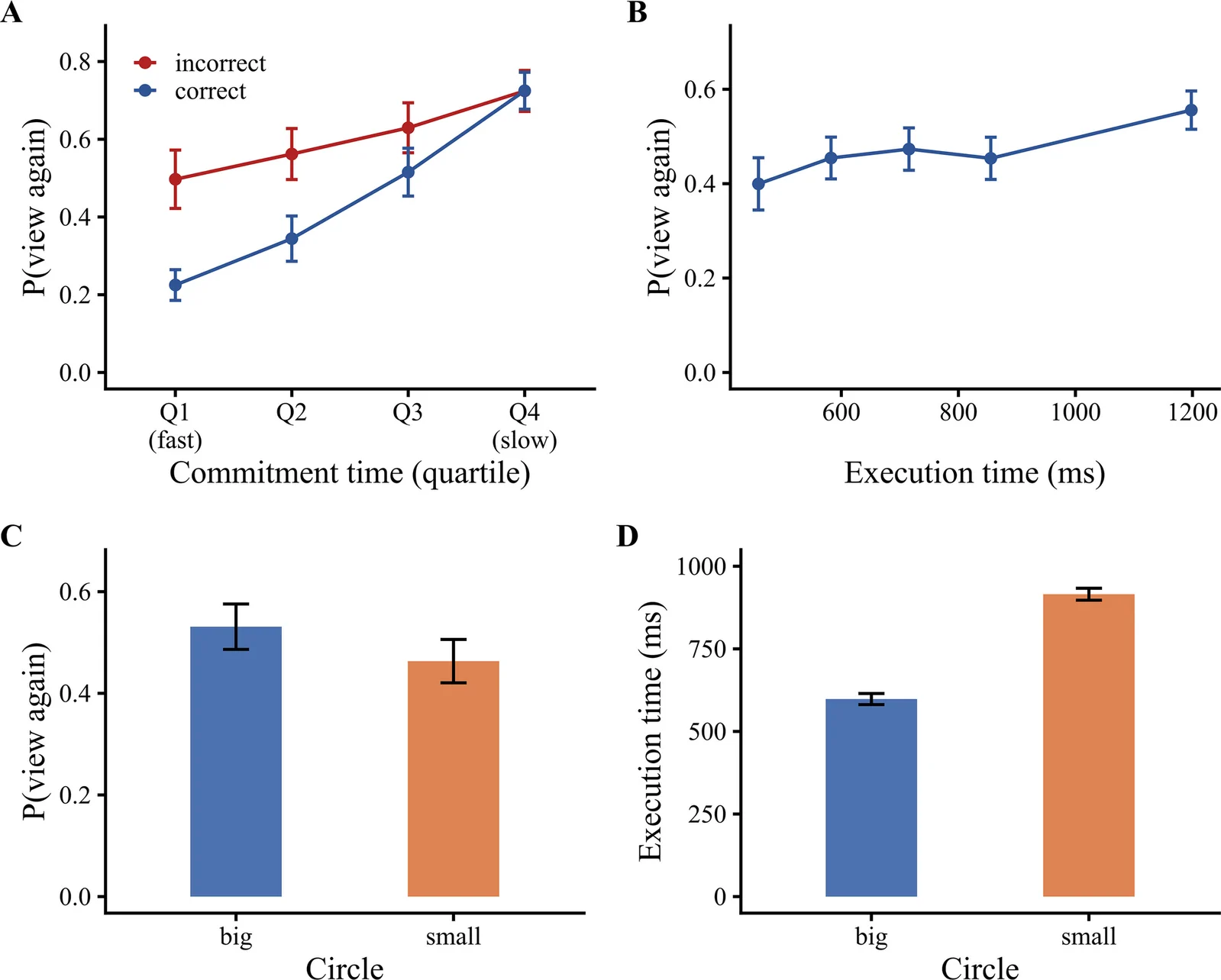
(**A**) Observed probability of requesting to view the stimulus again as a function of commitment-time quartile (Q1 = fastest, Q4 = slowest), shown separately for initially correct (blue) and incorrect (red) responses. (**B**) View-again probability increased with execution time (points represent execution-time quintiles). (**C**) View-again probability by circle size, adjusted for execution time: view-again rates were equated across execution-time quintiles within each participant before averaging (see *Methods*), isolating the effect of circle size from its influence on movement duration. (**D**) Execution time was longer for small than large circles, reflecting the greater precision demands of reaching smaller targets. Points and bars show means across participants; error bars represent standard errors

The same analyses were conducted on ET (et ~ accuracy*circle*position + (1 + accuracy*circle*position | subject)). Accuracy had no credible influence on ET (β = −3.98, CrI [−8.90, 0.85]). By contrast, circle had a large impact (β = 134.26, CrI [126.59, 141.58]), with ET being longer for small (*M* = 916 ms, *SD* = 248 ms) than big circles (*M* = 598 ms, *SD* = 210 ms), indicating that movement time increased with the precision demands of the response, as expected from Fitts's law (Fig. 2D and OSM Tables 2S and 3S).

An effect of position was also observed, with circles at the bottom leading to slower ET than the across-position average (β = 8.65, CrI [1.34, 16.61]). Follow-up comparisons with emmeans showed that bottom circles (*M* = 764, *SD* = 283) elicited slower ET than top circles (*M* = 752, *SD* = 284; contrast estimate = 13.65, CrI [0.39, 28.1]). Thus, moving the mouse cursor toward the body (to reach the bottom circles) takes somewhat longer than moving it away from the body (to reach the top circles).

The overall RTs (CT + ET) were also analyzed. In brief, RTs were shorter for big than small circles and shorter for correct than incorrect responses, mirroring the effects observed separately on CT and ET (OSM Table 4S).

Overall, the size of the response circles affected the time needed to *confirm* the perceptual response (i.e., ET), without influencing the time to *commit* to a perceptual decision (i.e., CT).

### Accuracy

We analyzed accuracy with a model including commitment time, circle, and position, and their interactions as fixed effect (accuracy ~ circle*position*ct + (1 + circle*position*ct | subject)). ET was not used as a predictor because it occurred after the perceptual decision (i.e., after the participant has committed to move left or right) and thus cannot determine accuracy. In addition, the analyses presented above showed that accuracy did not predict ET.

We observed that accuracy decreased with the increase of commitment time (odds ratio = 0.28, CrI [0.20, 0.40]). Importantly, circle size did not influence accuracy (odds ratio = 0.93, CrI [0.85, 1.01]). We observed evidence only for an interaction between circle and position (odds ratio = 0.89, CrI [0.80, 0.99]). Follow-up comparisons showed that accuracy was lower for small than big circles at the middle position (odds ratio = 0.68, CrI [0.51, 0.88]; observed average accuracy: 0.68 (small) vs. 0.76 (big)), whereas no credible difference emerged at the bottom or top positions (OSM Tables 5S and 6S). This change in accuracy did not modulate information seeking (see below).

#### Information seeking

We analyzed information seeking with the following model: view-again ~ accuracy*circle*position*(ct + et) + (1 + accuracy*circle*position*(ct + et) | subject). We included CT and ET as separate predictors rather than overall RT, since the analyses above showed that they reflect distinct processes. This allowed us to assess the effect of circle size independent of these timing variables, which are themselves associated with circle size. Analyses using overall RT yielded consistent results (OSM Table 7S).

Table 1 reports only the effects showing strong evidence (see Table 8S, OSM, for the full results). The size of the response circle guided information seeking (odds ratio = 0.84, CrI [0.74, 0.94], see Fig. 2C), with a higher tendency to seek additional evidence with big circles (adjusted *M* = 0.53, *SD* = 0.22) relative to small circles (adjusted *M* = 0.46, *SD* = 0.21).^3^

Moreover, information seeking decreased with correct responses (adjusted *M* = 0.46, *SD* = 0.23) compared to errors (adjusted *M* = 0.60, *SD* = 0.26; odds ratio = 0.67, CrI [0.58, 0.76]), and it increased with longer commitment times (odds ratio = 81.23, CrI [22.75, 327.74]). These results suggest that information-seeking behavior does indeed reflect confidence estimations, with participants seeking additional information when incorrect and slow. There was also evidence that longer ET led to a higher tendency to view the stimulus again (odds ratio = 4.10, CrI [2.49, 6.87], see Fig. 2B).

Finally, we observed two reliable interactions involving commitment time. First, the accuracy and CT interaction (odds ratio = 2.35, CrI [1.52, 3.57]) indicated that view-again responses were higher after incorrect than correct responses when commitment was fast, but this difference was strongly attenuated as commitment time increased (see Fig. 2A). Second, the interaction between circle, position, and commitment time (odds ratio = 0.57, CrI [0.33, 0.96]) indicated that commitment time predicted view-again responses differently for big and small circles at the middle target position only. Given that this interaction was not observed by Sanchez et al. (2024a, 2024b), and emerged for only one of the three positions without a systematic pattern, we refrain from interpreting it pending replication.

In sum, information seeking strongly correlated with both accuracy and commitment time, in a way that mirrors confidence estimations. Circle size and ET modulated information seeking in opposite directions: small circles *reduced* view-again responses, whereas longer ETs *increased* them.

#### Metacognitive efficiency, change of mind, and final accuracy

We assessed whether circle size affected metacognitive efficiency by estimating M-ratio (Maniscalco & Lau, 2012) and the relative meta-information (RMI; Meyen et al., 2025). In short, we found that M-ratio was higher for small (*M* = 1.13, *SD* = 0.63) than for big circles (*M* = 0.76, *SD* = 0.48; estimate = 0.33, CrI [0.06, 0.60]), whereas the difference in RMI, although in the same direction, was not credible (*M* small = 0.23, *SD* = 0.15; *M* big = 0.20, *SD* = 0.11; estimate = 0.029, CrI [−0.030, 0.091]). We interpret this pattern as suggestive evidence of higher metacognitive efficiency with small response circles (see OSM  for a full report on these analyses).

We also analyzed final accuracy on trials where participants asked to view the stimulus again, and changes of mind (i.e., trials on which after re-viewing participants judged their initial response as incorrect). Briefly, we observed that final accuracy (*M* = 0.74, *SD* = 0.10) was higher than initial accuracy (*M* = 0.61, *SD* = 0.07; odds ratio for after vs. before viewing the stimulus again = 1.88, CrI [1.54, 2.34]). In addition, changes of mind occurred more often after incorrect (*M* = 0.57, *SD* = 0.19) than correct initial responses (*M* = 0.16, *SD* = 0.09; odds ratio = 0.34, CrI [0.26, 0.43]). Circle size credibly predicted neither final accuracy nor changes of mind. Accordingly, circle size influenced whether participants sought additional evidence, but not the consequences of that evidence (see OSM Tables 9S and 10S for these analyses).

## Discussion

We investigated whether the motor cost associated with reporting a perceptual decision influences information seeking, a key feature of metacognitive evaluation. Previous research showed that motor signals contribute to retrospective confidence (Gajdos et al., 2019; Pereira et al., 2020; Sanchez 2024a, 2025; Turner et al., 2021). However, it remained unclear whether such action-induced changes in confidence exert a functional impact on subsequent behavior.

We manipulated the motor precision of perceptual responses by varying the size of the circles that participants needed to reach to report a decision (cf. Sanchez et al., 2024a). It is well known that reaching smaller targets requires higher precision in visuo-motor coordination than reaching big targets (Beamish et al., 2006; Fitts, 1954). As expected, we observed that reaching small circles resulted in longer execution times, whereas commitment time and accuracy remained unaffected. These findings argue against an account in which our manipulation alters confidence early, during the decision itself: such an account would predict an effect of circle size on commitment time, which we did not observe. Motor-related signals more likely contribute to confidence around or after response execution.

Regarding information seeking, we found strong evidence that participants tended to seek additional evidence following errors and slow decisions, i.e., on trials where uncertainty and hesitation were higher. This pattern is consistent with the literature on both information seeking and confidence, which shows that errors and slow decisions are associated with lower confidence and a higher tendency to seek additional evidence (Desender et al., 2018; Kiani et al., 2014; Pescetelli et al., 2021; Sanchez et al., 2024b).

In line with our main hypothesis, higher motor precision led to a reduced tendency to seek information without impacting perceptual accuracy. This result mirrors earlier studies observing that fine motor demands inflate confidence ratings (Sanchez et al., 2024a; Turner et al., 2021), and extends them by demonstrating that motor information also guides adaptive behavior. In addition, we found suggestive evidence that metacognitive efficiency was higher in trials requiring higher motor precision. However, this finding should be interpreted with caution and needs replication.

The fact that circle size influenced information seeking even after accounting for accuracy, commitment time, and execution time indicates that motor precision provides information for confidence computations that is independent of sensory information (i.e., the actual Gabor’s orientation) underlying the decision. One might object that, because small circles were associated with longer execution times, participants could have accumulated more sensory evidence during these longer movements, thereby increasing their confidence and (weakly) improving their metacognitive efficiency. However, circle size and execution time modulated information seeking in opposite directions: controlling for execution time, small circles reduced the proportion of view-again responses, whereas longer execution times increased it. This pattern is incompatible with an evidence-accumulation account: if the confidence boost for small circles arose from accumulating evidence over a longer movement, then longer execution times should themselves have been associated with less information seeking (i.e., higher confidence), whereas we observed the opposite effect.

However, the precise mechanism through which motor information influences confidence and information seeking remains unclear. Overall these findings are compatible with the Bayesian framework of metacognition proposed by Fleming and Daw (2017), in which confidence integrates multiple sources of information beyond sensory evidence. According to this view, motor-related signals feed confidence over and above sensory evidence and both sources of information are combined into a single confidence estimate. Within this framework, several explanations can be proposed regarding the way motor information may be used for metacognitive evaluations. First, it has been suggested that confidence evaluations likely rely on heuristics that employ cues linked to the quality of decision making (cf. Ackerman, 2019; Allen et al., 2016; Kiani et al., 2014; Maniscalco et al., 2016; Shekhar & Rahnev, 2024; Van Marcke et al., 2024). One notion that might be relevant here is the idea of commitment: people tend to feel more committed to correct than incorrect decisions. According to this view, decisions requiring a more controlled and effortful movement may retrospectively feel more “committed” and thus more likely to be correct. In other words, the effect we observed could be attributed to a “motoric sunk cost”: a greater investment of motor resources in implementing a decision makes it appear more likely to be accurate (cf. Turner et al., 2021; but see Hagura et al., 2023). This explanation can account for the general bias in confidence or information seeking associated with small circles (cf. Sanchez et al., 2024a); however, it is less clear how it would explain the weak improvement in metacognitive performance we observed.

A second possible explanation is that responses requiring greater precision entail stronger engagement of action control and action monitoring processes (Gajdos et al., 2019; Sanchez et al., 2024a, 2025). Such enhanced monitoring may have two consequences. On the one hand, it might bias confidence (“I monitored my response more carefully, therefore I must be correct”). On the other hand, it might improve access to internal signals associated with decision quality, thereby enhancing metacognitive performance. This interpretation is supported by evidence that perceptual decision signals are present in neural circuits involved in motor actions (Freedman & Assad, 2011; Gold & Shadlen, 2007; Romo et al., 2004; Shadlen & Newsome, 2001), and perceptual decision-making models suggest that sensory evidence is accumulated within motor effector areas (Gold & Shadlen, 2007; Vickers, 1979). Thus, more intensive monitoring of an action may enhance access to the quality of the underlying decision. In line with this interpretation, recent findings indicate that overt motor responses can provide a more robust read-out of decision evidence than confidence judgments made without overt perceptual decision (Pereira et al., 2020).

Taken together, our findings suggest that either directly or indirectly the action system contributes to metacognitive evaluation that participants use to regulate their behavior. Motor-induced confidence changes are therefore not epiphenomenal: they exert a direct functional influence on information seeking, a behavioral strategy central to adaptive decision making.

## Supplementary Information

Below is the link to the electronic supplementary material.

Supplementary file1 (DOCX 129 KB)

## Data Availability

Data are available on Zenodo: https://zenodo.org/records/23235482

## Footnotes

1 This average is based on the age of 23 subjects. One adult participant did not report their age.

2 Unless explicitly indicated, descriptive statistics such as averages and standard deviations reflect the actual mean and spread of the observed data.

3 Adjusted means were computed by equating conditions on the timing variable through which they are confounded. For circle size, trials were grouped into five ET quintiles and view-again rates averaged across quintiles within each participant; this adjustment was necessary because small circles substantially lengthened ET (*M* = 916 ms vs. 598 ms for large circles), and slower trials were themselves associated with more view-again requests, so that unadjusted means conflate the effect of circle size with that of its consequence on movement duration. For accuracy, the same procedure was applied using CT quintiles, since incorrect responses were preceded by longer CTs. Adjusted means therefore illustrate each effect at matched response latencies. CT did not differ between circle sizes and was therefore not included in the size standardization, and both timing variables are controlled for in the models.

## Code availability

Experiment and analyses scripts are available on Zenodo: https://zenodo.org/records/23235482
