# Supplementary material for: Motor precision shapes information seeking in perceptual decisions
Source: Psychon Bull Rev. 2026 Nov 6;33(8):271. doi: 10.3758/s13423-026-03038-8 (PMC13743573; doi:10.3758/s13423-026-03038-8)
Supplement: Supplementary file 1 [file 13423_2026_3038_MOESM1_ESM.docx]

**Supplementary Information for**

**Motor precision shapes information seeking in perceptual decisions**

Alessandra Loretani ^1, 2^, Claire Bédé ^1^, Thibaut Gajdos Preuss ^*^ ^3^, Andrea Desantis ^* 1, 2, 4^

^1^ Département Traitement de l’Information et Systèmes, ONERA, Salon-de-Provence, France

^2^ Institut de Neurosciences de la Timone (UMR 7289), CNRS and Aix-Marseille Université, Marseille, France

^3^ Centre de Recherche en Psychologie et Neurosciences (UMR 7077), CNRS, Aix Marseille Université, France

^4^ Integrative Neuroscience and Cognition Center (UMR 8002), CNRS and Université Paris Cité, Paris, France

* Co-senior authors

Corresponding author: Andrea Desantis, [aerdna.desantis@gmail.com](mailto:aerdna.desantis@gmail.com)

**Bayesian analysis**

In all Bayesian analyses we used weakly informative priors (Normal(0,10)) for regression parameters, and performed 4000 iterations (burn-in period: 1000). All models converged (all R̂ ≤ 1.01), with no divergent transitions and adequate effective sample sizes for all parameters (bulk- and tail-ESS > 400). Contrasts were coded using sum contrasts and were as follows: Accuracy: error = -1, correct = 1; Circle: small = 1, big = -1; Position: 1 if down, 0 if middle, -1 if up.

**Results**

**Commitment time.** The Table 1S summarizes hierarchical Bayesian regression results of the model predicting commitment time from accuracy, circle, and position. For each predictor, the table provides the posterior mean estimate and the corresponding 95% credible interval based on the posterior distribution.

**Table 1S:** Results of hierarchical Bayesian regression on commitment time.

|  | **commitment time (ms)** | |
| --- | --- | --- |
| *Predictors* | *Estimates* | *CI (95%)* |
| Intercept | 833.72 | 793.49 – 872.60 |
| accuracy_correct | -19.95 | -25.73 – -14.74 |
| circle_small | 2.44 | -1.76 – 6.63 |
| position_bottom | -0.47 | -8.38 – 7.64 |
| position_middle | -5.80 | -13.40 – 1.72 |
| accuracy_correct × circle_small | -2.67 | -6.70 – 1.36 |
| accuracy_correct × position_bottom | 5.01 | -1.33 – 11.41 |
| accuracy_correct × position_middle | -1.73 | -11.05 – 8.05 |
| circle _small × position_bottom | -5.60 | -12.32 – 1.01 |
| circle _small × position_middle | -1.24 | -7.24 – 4.82 |
| accuracy_correct × circle _small × position_bottom | 3.78 | -1.98 – 9.61 |
| accuracy_correct × circle _small × position_middle | 2.80 | -4.46 – 10.09 |
| N _subject_ | 24 | |
| Observations | 4508 | |

**Figure 1S.** Commitment time as a function of response accuracy (left) and circle size (right). Points show mean commitment time across participants computed from the observed data; error bars represent within-participant standard errors (Cousineau–Morey method). Commitment time was shorter for accurate responses compared to errors. No evidence of an effect of circle size on commitment time was observed.

**Execution time.** The Table 2S summarizes hierarchical Bayesian regression results of the model predicting execution time from accuracy, circle, and position. For each predictor, the table provides the posterior mean estimate and the corresponding 95% credible interval based on the posterior distribution. The contrast isolating the bottom position of the three-way interaction between accuracy circle and position, showed a 95% credible interval that excluded zero (β = -5.60, 95% CrI [-10.88, -0.07]), suggesting that the accuracy and circle interaction at the bottom position differed from the accuracy and circle interaction averaged across positions. However, this effect was not supported by follow-up comparisons using emmeans (see Table 3S in the Supplementary Material). Similarly, leave-one-out cross-validation revealed no meaningful predictive difference between the full model (et ~ accuracy*circle*position + (1 + accuracy*circle*position | subject)) and a reduced model without the three-way term (et ~ (accuracy + circle + position)^2 + (1 + (accuracy + circle + position)^2 | subject); Delta elpd (expected log pointwise predictive density) = −2.3, SE = 2.7). The three-way interaction was therefore not further interpreted.

**Table 2S:** Results of hierarchical Bayesian regression on execution time.

|  | **execution time (ms)** | |
| --- | --- | --- |
| *Predictors* | *Estimates* | *CI (95%)* |
| Intercept | 757.40 | 728.80 – 786.84 |
| accuracy_correct | -3.98 | -8.90 – 0.85 |
| circle_small | 134.26 | 126.59 – 141.58 |
| position_bottom | 8.65 | 1.34 – 16.61 |
| position_middle | -3.71 | -11.18 – 3.73 |
| accuracy_correct × circle_small | -0.20 | -4.19 – 3.80 |
| accuracy_correct × position_bottom | -2.53 | -8.16 – 2.92 |
| accuracy_correct × position_middle | 2.31 | -3.15 – 7.66 |
| circle_small × position_bottom | 3.36 | -2.19 – 8.99 |
| circle_small × position_middle | -6.00 | -11.81 – 0.08 |
| accuracy_correct × circle_small × position_bottom | -5.60 | -10.88 – -0.07 |
| accuracy_correct × circle_small × position_middle | 2.73 | -2.86 – 8.26 |
| N _subject_ | 24 | |
| Observations | 4508 | |

**Table 3S:** Follow-up contrasts with emmans of the three-way interaction accuracy x circle x position (execution time)

| *Position* | *Contrast* | *Estimate* | *CI..95..* |
| --- | --- | --- | --- |
| down | accuracy (correct - incorrect) x circle (big - small) | -23.38 | -49.98 – 3.3 |
| mid | accuracy (correct - incorrect) x circle (big - small) | 10.12 | -16.26 – 39.42 |
| up | accuracy (correct - incorrect) x circle (big - small) | 10.39 | -18.04 – 37 |

**Response times.** Similarly to Sanchez, Courant et al. (2024), we assessed the impact of accuracy (correct, incorrect responses), circle (big, small circles), position (top, middle, bottom), and their interaction on response times (RT; the overall response latency). The same factors were used to estimate correlated by-participant random slopes (rt ~ accuracy * circle * position + (1 + accuracy * size * position | subject)). We observed strong evidence that RT were shorter when participants reached big circles (M = 1422 ms, SD = 387) than small circles (M= 1746 ms, SD = 399; β = 146.46, SD = 4.61, 95% CrI [136.92, 155.62]). As we observed above this effect was particularly accounted by the execution time. Response times also differed as a function of accuracy, with shorter RTs for correct (M = 1536 ms, SD = 407) than for incorrect responses (M = 1701 ms, SD = 444; β = -28.60, SD = 4.81, 95% CrI [-39.08, -19.24]), reflecting the relationship between commitment time and accuracy observed above. In addition, the results showed that circles in the middle position exhibited shorter response times than the average across the three positions (β = -14.37, SD = 6.32, 95% CrI [-27.06, -1.78]). Follow-up analyses with emmeans comparing each position against the other showed no credible differences of RT between positions (down - middle: 22.16 ms, 95% CrI [−1.03, 44.49]; down - up: 1.04 ms, 95% CrI [−31.24, 33.53]; middle - up: −21.04 ms, 95% CrI [−47.65, 4.92]); accordingly, this effect was not further interpreted. None of the other effects showed strong credible differences: all 95% credible intervals included zero (see Table 4S).

**Table 4S:** Results of hierarchical Bayesian regression on response time.

|  | **response time (ms)** | |
| --- | --- | --- |
| *Predictors* | *Estimates* | *CI (95%)* |
| Intercept | 1591.30 | 1523.58 – 1659.90 |
| accuracy_correct | -28.60 | -39.08 – -19.24 |
| circle_small | 146.46 | 136.92 – 155.62 |
| position_bottom | 7.74 | -8.50 – 24.54 |
| position_middle | -14.37 | -27.06 – -1.78 |
| accuracy_correct × circle_small | -1.01 | -8.20 – 6.10 |
| accuracy_correct × position_bottom | 1.48 | -8.78 – 11.53 |
| accuracy_correct × position_middle | 4.62 | -8.76 – 17.91 |
| circle_small × position_bottom | -2.62 | -12.84 – 7.36 |
| circle_small × position_middle | -8.81 | -19.48 – 1.60 |
| accuracy_correct × circle_small × position_bottom | 0.05 | -9.93 – 9.87 |
| accuracy_correct × circle_small × position_middle | 4.81 | -6.43 – 15.57 |
| N _subject_ | 24 | |
| Observations | 4508 | |

**Accuracy.** The Table 5S below summarizes hierarchical Bayesian regression results of the model predicting accuracy from circle, position and commitment time.

**Table 5S:** Results of hierarchical Bayesian regression on accuracy.

|  | **accuracy** | |
| --- | --- | --- |
| *Predictors* | *Odds Ratios* | *CI (95%)* |
| Intercept | 2.48 | 2.12 – 2.92 |
| circle_small | 0.93 | 0.85 – 1.01 |
| position_bottom | 0.98 | 0.88 – 1.10 |
| position_middle | 1.06 | 0.95 – 1.18 |
| commitment time | 0.28 | 0.20 – 0.40 |
| circle_small × position_bottom | 1.05 | 0.94 – 1.16 |
| circle_small × position_middle | 0.89 | 0.80 – 0.99 |
| circle_small × commitment time | 0.90 | 0.70 – 1.17 |
| position_bottom × commitment time | 1.08 | 0.75 – 1.53 |
| position_middle × commitment time | 1.26 | 0.86 – 1.87 |
| circle_small × position_bottom × commitment time | 1.13 | 0.81 – 1.56 |
| circle_small × position_middle × commitment time | 1.03 | 0.73 – 1.47 |
| N _subject_ | 24 | |
| Observations | 4508 | |

| **Table 6S:** Mean observed accuracy by target position and circle size | | |
| --- | --- | --- |
| *Position* | *Big circles* | *Small circles* |
| down | 0.707 (n = 750) | 0.697 (n = 752) |
| mid | 0.756 (n = 751) | 0.679 (n = 760) |
| up | 0.702 (n = 745) | 0.696 (n = 750) |

**View again.** The Table 7S presents hierarchical Bayesian regression table predicting “view again” judgments from accuracy, circle, position, and response time. Response times were mean-centered.

**Table 7S:** Results of hierarchical Bayesian regression on view again.

|  | **View again** | |
| --- | --- | --- |
| *Predictors* | *Odds Ratios* | *CI (95%)* |
| Intercept | 1.24 | 0.66 – 2.39 |
| accuracy_correct | 0.65 | 0.58 – 0.73 |
| circle_small | 0.70 | 0.61 – 0.79 |
| position_bottom | 0.99 | 0.86 – 1.14 |
| position_middle | 1.04 | 0.91 – 1.20 |
| response time | 12.11 | 6.70 – 22.86 |
| accuracy_correct × circle_small | 0.83 | 0.76 – 0.92 |
| accuracy_correct × position_bottom | 1.04 | 0.91 – 1.21 |
| accuracy_correct × position_middle | 1.03 | 0.89 – 1.18 |
| circle_small × position_bottom | 0.93 | 0.82 – 1.07 |
| circle_small × position_middle | 0.95 | 0.83 – 1.08 |
| accuracy_correct × response time | 1.77 | 1.35 – 2.30 |
| circle_small × response time | 0.61 | 0.42 – 0.86 |
| position_bottom × response time | 1.20 | 0.83 – 1.74 |
| position_middle × response time | 1.06 | 0.75 – 1.52 |
| accuracy_correct × circle_small × position_bottom | 1.01 | 0.89 – 1.15 |
| accuracy_correct × circle_small × position_middle | 1.07 | 0.94 – 1.23 |
| accuracy_correct × circle_small × response time | 0.80 | 0.63 – 1.03 |
| accuracy_correct × position_bottom × response time | 0.85 | 0.60 – 1.20 |
| accuracy_correct × position_middle × response time | 0.91 | 0.63 – 1.30 |
| circle_small × position_bottom × response time | 0.90 | 0.64 – 1.26 |
| circle_small × position_middle × response time | 0.95 | 0.68 – 1.33 |
| accuracy_correct × circle_small × position_bottom × response time | 0.94 | 0.67 – 1.31 |
| accuracy_correct × circle_small × position_middle × response time | 0.85 | 0.60 – 1.21 |
| N _subject_ | 24 | |
| Observations | 4508 | |

**View again.** The Table 8S presents hierarchical Bayesian regression table predicting “view again” judgments from accuracy, circle, position, commitment and execution time. Timing predictors were mean-centered.

**Table 8S:** Results of hierarchical Bayesian regression on view again.

|  | **View again** | |
| --- | --- | --- |
| *Predictors* | *Odds Ratios* | *CI (95%)* |
| Intercept | 1.52 | 0.77 – 2.96 |
| accuracy_correct | 0.67 | 0.58 – 0.76 |
| circle_small | 0.84 | 0.74 – 0.94 |
| position_bottom | 1.05 | 0.89 – 1.24 |
| position_middle | 0.95 | 0.82 – 1.12 |
| execution time | 4.10 | 2.49 – 6.87 |
| commitment time | 81.23 | 22.75 – 327.74 |
| accuracy_correct × circle_small | 0.90 | 0.80 – 1.01 |
| accuracy_correct × position_bottom | 1.05 | 0.89 – 1.23 |
| accuracy_correct × position_middle | 1.06 | 0.90 – 1.24 |
| circle_small × position_bottom | 0.93 | 0.79 – 1.09 |
| circle_small × position_middle | 0.95 | 0.81 – 1.12 |
| accuracy_correct × execution time | 1.09 | 0.71 – 1.65 |
| accuracy_correct × commitment time | 2.35 | 1.52 – 3.57 |
| circle_small × execution time | 0.60 | 0.33 – 1.04 |
| circle_small × commitment time | 0.80 | 0.54 – 1.16 |
| position_bottom × execution time | 1.37 | 0.72 – 2.64 |
| position_middle × execution time | 1.03 | 0.56 – 1.90 |
| position_bottom × commitment time | 1.08 | 0.62 – 1.83 |
| position_middle × commitment time | 0.85 | 0.50 – 1.42 |
| accuracy_correct × circle_small × position_bottom | 1.01 | 0.86 – 1.19 |
| accuracy_correct × circle_small × position_middle | 1.06 | 0.90 – 1.24 |
| accuracy_correct × circle_small × execution time | 0.77 | 0.50 – 1.20 |
| accuracy_correct × circle_small × commitment time | 0.84 | 0.58 – 1.22 |
| accuracy_correct × position_bottom × execution time | 0.79 | 0.44 – 1.46 |
| accuracy_correct × position_middle × execution time | 0.91 | 0.49 – 1.66 |
| accuracy_correct × position_bottom × commitment time | 0.86 | 0.50 – 1.47 |
| accuracy_correct × position_middle × commitment time | 1.00 | 0.60 – 1.65 |
| circle_small × position_bottom × execution time | 0.68 | 0.37 – 1.22 |
| circle_small × position_middle × execution time | 1.64 | 0.91 – 3.02 |
| circle_small × position_bottom × commitment time | 1.14 | 0.69 – 1.98 |
| circle_small × position_middle × commitment time | 0.57 | 0.33 – 0.96 |
| accuracy_correct × circle_small × position_bottom × execution time | 0.90 | 0.50 – 1.64 |
| accuracy_correct × circle_small × position_middle × execution time | 0.66 | 0.35 – 1.26 |
| accuracy_correct × circle_small × position_bottom × commitment time | 0.99 | 0.58 – 1.75 |
| accuracy_correct × circle_small × position_middle × commitment time | 1.04 | 0.62 – 1.83 |
| N _subject_ | 24 | |
| Observations | 4508 | |

**Metacognitive efficiency**

We assessed whether circle size affected metacognitive efficiency by estimating meta-d′/d′ (M-ratio; Maniscalco & Lau, 2012) and the relative meta-information (RMI; Meyen et al., 2025), a bias-reduced, assumption-free information-theoretic measure of metacognitive efficiency. These measures were calculated separately for each participant and circle, treating view-again responses as a binary confidence rating (view again = low confidence). For each measure, we then computed within-participant difference scores between conditions (small - big circle) and modeled them with a Bayesian intercept-only regression using a Student-t likelihood and a weakly informative normal(0, 1) prior on the intercept. In these models, the intercept directly estimates the mean paired difference between conditions. M-ratio was credibly higher for small (M = 1.13, SD = 0.63) than for big circles (M = 0.76, SD = 0.48; estimate = 0.33, 95% CrI [0.06, 0.60]), whereas the difference in RMI, although in the same direction, was not credible (M small = 0.23, SD = 0.15; M big = 0.20, SD = 0.11; estimate = 0.029, 95% CrI [−0.030, 0.091]). We interpret this pattern as suggestive evidence of higher metacognitive efficiency with small response circles.

**Final accuracy.** On trials where participants requested to view the stimulus again, we derived the hypothetical accuracy of a revised decision. After the second presentation, participants judged whether their initial response was correct or not (they did not explicitly report the orientation again). This judgment logically determines a revised decision: judging the initial response correct is equivalent to maintaining it, and judging it incorrect is equivalent to selecting the alternative orientation. The final accuracy (i.e., the accuracy of this revised decision) was then compared to initial accuracy. For these analyses we did not include circles position as a predictor. This decision was motivated by two aspects. First, across all previous analyses, position exerted marginal influences on the results. Second, these analyses were conducted on the subset of view-again trials only, which substantially reduced the number of observations. Hence, omitting position (a three-level factor whose full interaction with the other predictors would have added numerous parameters) kept the models better identified given the smaller sample.

A first model assessed whether final accuracy on view-again trials was higher than initial accuracy (accuracy ~ stage + (1 + stage | subject), where stage denotes the moment of the decision, i.e., before vs. after viewing the Gabor again). The analysis confirmed that seeking additional evidence was beneficial: accuracy of the final decision (M = 0.74, SD = 0.10) was higher than that of the initial decision (M = 0.61, SD = 0.07; odds ratio = 1.88, 95% CrI [1.54, 2.34]).

A second model assessed the final accuracy on view-again trials as a function of initial accuracy, circle size, commitment time, and execution time:

final accuracy ~ accuracy * size * (commitment time + execution time) + (1+ accuracy * size * (commitment time + execution time) | subject))

Final decisions were far more often correct when the initial response had been correct than when it had been incorrect (odds ratio = 2.44, 95% CrI [1.83, 3.28]): participants maintained approximately 88% of their correct initial decisions but recovered only about 55% of their erroneous ones, indicating that the overall benefit of re-viewing arose primarily from maintaining decisions that were already correct rather than from recovering errors. Commitment time interacted with initial accuracy (odds ratio = 0.38, 95% CrI [0.25, 0.61]): longer commitment times increased the accuracy of the final decision when the initial response had been incorrect, but decreased it when the initial response had been correct. This pattern indicates that initial hesitation made participants more prone to reverse their decision irrespective of whether reversal was warranted, rather than improving their ability to identify which decisions needed revising. Neither circle size nor any of its interactions credibly predicted the accuracy of the final decision (all 95% CrIs included 1), indicating that the benefit of viewing the stimulus again was comparable across motor demands. Accordingly, this suggests that circle size influenced whether participants sought additional evidence, but not the consequences of that evidence.

**Table 9S:** Results of hierarchical Bayesian regression on final accuracy.

|  | **Final accuracy** | |
| --- | --- | --- |
| *Predictors* | *Odds Ratios* | *CI (95%)* |
| Intercept | 2.95 | 2.26 – 3.84 |
| accuracy_correct | 2.44 | 1.83 – 3.28 |
| size_small | 0.91 | 0.75 – 1.06 |
| commitment time | 0.94 | 0.61 – 1.47 |
| execution time | 0.71 | 0.43 – 1.18 |
| accuracy_correct × size_small | 1.06 | 0.90 – 1.25 |
| accuracy_correct × commitment time | 0.38 | 0.25 – 0.61 |
| accuracy_correct × execution time | 0.63 | 0.36 – 1.16 |
| size_small × commitment time | 1.23 | 0.81 – 1.90 |
| size_small × execution time | 1.49 | 0.86 – 2.67 |
| accuracy_correct × size_small × commitment time | 1.00 | 0.67 – 1.50 |
| accuracy_correct × size_small × execution time | 0.84 | 0.51 – 1.35 |
| N _subject_ | 24 | |
| Observations | 2096 | |

**Change of mind.** We also examined changes of mind, notably trials in which participants, after viewing the stimulus again, judged their initial response to have been incorrect (see Table 9S). On average, participants changed their mind on 32% of view-again trials. To further explore these trials we fitted a Bayesian mixed-effects logistic regression predicting change of mind from initial accuracy, circle, commitment time, and execution time (change of mind ~ accuracy * circle * (commitment time + execution time) + (1 + accuracy * circle * (commitment time + execution time))). In addition, changes of mind occurred more often after incorrect (M = 0.57, SD = 0.19) than correct initial responses (M = 0.16, SD = 0.09), odds ratio = 0.34, 95% CrI [0.26, 0.43]). Commitment time further increased the tendency to revise initial decision, with change of mind increasing with the increase of commitment time (odds ratio = 2.59, 95% CrI [1.62, 3.97]). Neither circle size nor any of its interactions credibly predicted changes of mind (all 95% CrIs included 1). Accordingly, as for the final accuracy, this suggests that circle size influenced whether participants sought additional evidence, but not the consequences of that evidence.

**Table 10S:** Results of hierarchical Bayesian regression on Change of mind.

|  | **Change of mind** | |
| --- | --- | --- |
| *Predictors* | *Odds Ratios* | *CI (95%)* |
| Intercept | 0.41 | 0.31 – 0.55 |
| accuracy_correct | 0.34 | 0.26 – 0.43 |
| size_small | 0.95 | 0.81 – 1.11 |
| commitment time | 2.59 | 1.62 – 3.97 |
| execution time | 1.57 | 0.86 – 2.84 |
| accuracy_correct × size_small | 1.10 | 0.94 – 1.32 |
| accuracy_correct × commitment time | 1.06 | 0.69 – 1.64 |
| accuracy_correct × execution time | 1.40 | 0.85 – 2.34 |
| size_small × commitment time | 1.00 | 0.66 – 1.48 |
| size_small × execution time | 1.21 | 0.73 – 2.00 |
| accuracy_correct × size_small × commitment time | 0.81 | 0.52 – 1.23 |
| accuracy_correct × size_small × execution time | 0.67 | 0.38 – 1.18 |
| N _subject_ | 24 | |
| Observations | 2096 | |
